# Multiple consensus trees: a method to separate divergent genes

**DOI:** 10.1186/1471-2105-14-46

**Published:** 2013-02-09

**Authors:** Alain Guénoche

**Affiliations:** 1 Institut de Mathématiques de Luminy, 163 Av. de Luminy, 13009 Marseille, France

## Abstract

**Background:**

It is generally admitted that the species tree cannot be inferred from the genetic sequences of a single gene because the evolution of different genes, and thus the gene tree topologies, may vary substantially. Gene trees can differ, for example, because of horizontal transfer events or because some of them correspond to paralogous instead of orthologous sequences. A variety of methods has been proposed to tackle the problem of the reconciliation of gene trees in order to reconstruct a species tree. When the taxa in all the trees are identical, the problem can be stated as a consensus tree problem.

**Results:**

In this paper we define a new method for deciding whether a unique consensus tree or multiple consensus trees can best represent a set of given phylogenetic trees. If the given trees are all congruent, they should be compatible into a single consensus tree. Otherwise, several consensus trees corresponding to divergent genetic patterns can be identified. We introduce a method optimizing the *generalized score*, over a set of tree partitions in order to decide whether the given set of gene trees is homogeneous or not.

**Conclusions:**

The proposed method has been validated with simulated data (random trees organized in three topological groups) as well as with real data (bootstrap trees, homogeneous set of trees, and a set of non homogeneous gene trees of 30 *E. Coli* strains; it is worth noting that some of the latter genes underwent horizontal gene transfers). A computer program, MCT - Multiple Consensus Trees, written in C was made freely available for the research community (it can be downloaded from http://bioinformatics.lif.univ-mrs.fr/consensus/index.html). It handles trees in a standard Newick format, builds three hierarchies corresponding to RF and QS similarities between trees and the greedy ascending algorithm. The generalized score values of all tree partitions are computed.

## Background

The comparison of gene trees and their assembling in a unique tree representing the species tree is a fundamental problem in phylogeny. A large variety of methods have been proposed to tackle the problem of the reconciliation of gene trees in order to reconstruct a species tree. A panel of methodological approaches can be found in [1] preceding the authors’ own method. When the taxa in all the trees are identical, the problem can be stated as a consensus tree problem.

Given a set of phylogenetic trees, summarizing them into a consensus tree implies the homogeneity of this tree set. According to an alignment of sequences from a set *X* of *n* taxa, each gene gives an unrooted *X*-tree [2]; *X* is the set of leaves, the internal nodes have degree 3 and the edges have a positive or null length. These trees can be established with several kinds of methods, distance, likelihood, or parsimony [3]. A bootstrapping approach [4] is used to evaluate the robustness of each gene tree, using a consensus methodology. At this level the homogeneity of the tree set is guaranteed. Then comes the second consensus tree problem. It consists in computing, from the gene tree set, denoted in the following as a *profile* of *mX*-trees, a global consensus tree summarizing them, i.e. producing one species *X*-tree. For this consensus tree, the homogeneity of the profile is questionable because of horizontal transfer events or because some genes can correspond to paralogous instead of orthologous alleles.

Here, we only deal with unrooted *X*-trees. Several consensus strategies can be used [5]. We focus on those proceeding the same way : an *X*-tree is considered as a set of bipartitions, each one corresponding to an internal edge of the tree, the external ones connecting the leaves to the tree. Any internal edge clearly separates two subsets of *X* having more than one element. Removing this edge creates a *split*, inducing a bipartition of *X*. The weight of each bipartition $P_{i}=X_{i}\cup X_{i}^{\prime }$ is the number *m*_*i*_of *X*-trees in the profile containing this bipartition.

Given a set of *X*-trees, 

• the strict consensus tree is only made of bipartitions common to all the trees (*m*_*i*_=*m*). This is a theoretical consensus leading to very unresolved trees (with very few internal edges).

• the majority consensus tree contains all the bipartitions that are present in a majority of trees (*m*_*i*_>*m*/2). The bipartitions are compatible with a tree structure that is, generally, incompletely resolved.

• the extended majority consensus tree contains all the majority bipartitions, as well as all those that are compatible with the previous ones, the edges being selected according to decreasing values of *m*_*i*_. This greedy consensus is the usual one, since it leads to the most resolved consensus trees.

• the Nelson consensus tree is made with the heaviest set of compatible bipartitions ($\underset{i}{\sum }m_{i}$ is maximum). It corresponds to finding a clique of maximum weight in a compatibility graph of the whole bipartition set, which is NP-hard [6].

### Example 1

**Figure 1:**
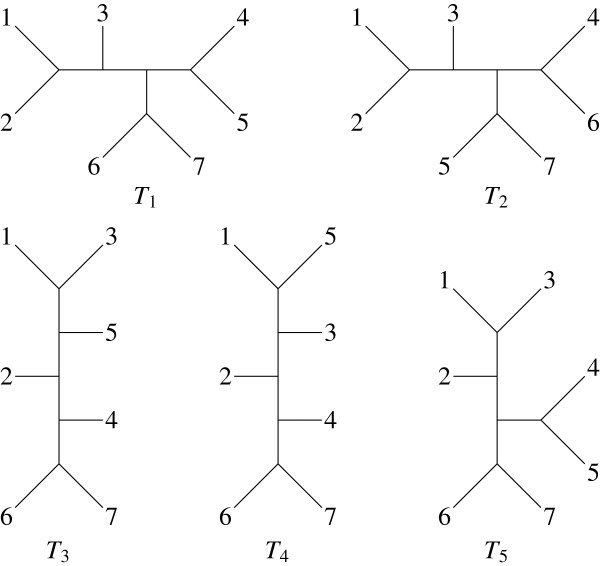
**Five *X*-trees of 7 leaves.**

**Figure 2:**
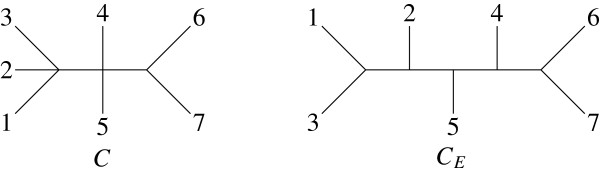
**The majority consensus tree *C *and an extended one *C***_***E***_**.**

Let *π* be the profile containing the 5 trees of Figure 1 : The computation of the consensus tree will first establish the complete set of bipartitions present in these trees, which is given in Table 1. The only majority bipartitions are the second and third, which are present in 3 and 4 trees respectively. They make the majority consensus tree *C* which can be extended with the 7-th and 9-th bipartitions to give the extended majority tree *C*_*E*_; both are drawn in Figure 2. *C* can also be extended with the 1-st and 9-th bipartitions to give a tree similar to *C*_*E *_exchanging leaves 2 and 3.

**Table 1:** **The whole set of bipartitions in the trees of Figure **1

	***T***_**1**_	***T***_**2**_	***T***_**3**_	***T***_**4**_	***T***_**5**_	**Bipartitions**
1	x	x				1 2 | 3 4 5 6 7
2	x	x			x	1 2 3 | 4 5 6 7
3	x		x	x	x	1 2 3 4 5 | 6 7
4	x				x	1 2 3 6 7 | 4 5
5		x				1 2 3 4 6 | 5 7
6		x				1 2 3 5 7 | 4 6
7			x		x	1 3 | 2 4 5 6 7
8			x	x		1 3 5 | 2 4 6 7
9			x	x		1 2 3 5 | 4 6 7
10				x		1 5 | 2 3 4 6 7

### How to assign a weight to the consensus tree?

In bootstrapping a gene sequence alignment, phylogeneticists are mainly interested in strong majority edges, i.e. edges that are in a great number of trees (generally at least 90%) indicating subsets of taxa derived from a common ancestor. This permits both analyzing the tree, to decide if it is a strong consensus, and also comparing it to another tree^a^. For these comparisons, and also for the consensus tree, we will focus only on the majority edges, those that are present in more than half the number of trees, because (i) differences based on minority edges would be less convincing, (ii) the majority consensus tree is a median of the profile, for the *Robinson-Foulds* distance [7] and (iii) it is unique.

The weight of an *X*-tree *A*, relative to profile *π*, is equal to the sum of the weights of its internal majority edges (those present in *m*_*i*_>*m*/2) corresponding to bipartitions *P*_*i*_. Let *A*_*m *_be the tree *A* restricted to its majority edges:

$$W_{\pi }(A)=\underset{P_{i}\in A_{m}}{\sum }w(P_{i})=\underset{P_{i}\in A_{m}}{\sum }m_{i}.$$

In the following, we only consider the majority consensus tree of *π*, i.e. the *X*-tree maximizing this weight function and containing only majority internal edges.

## Single or multiple consensus trees?

Now comes the main question : why summarize a set of *X*-trees with a single tree? When the profile is provided by a bootstrap procedure a single tree is expected ; adding noise to an alignment which is not certain, is to consider other potential alignments to test if they produce the same or a similar tree. But when several *X*-trees corresponding to different genes are examined, several consensus trees can be expected. If these genes are not congruent, i.e. reflecting the same evolutionary history, the unique consensus tree could contain few majority edges, with low weights, and even no internal edges at all, a star tree. Consequently, given a set of *X*-trees corresponding to several genes, one can ask if there is one consensus tree or several trees associated to subsets of genes having evolved differently and each having their own strong consensus.

This idea was first formulated by Maddison [8] with the Phylogenetic Islands concept based on pruning and regrafting of just one subtree. He observed that the consensus trees of the islands are different and with a better resolution than for the whole set. This principle has been extended by [9] who investigate several clustering procedures of a given tree set to compare only strict consensus, without indicating how to fix the number of clusters. More recently, [10] give a method to build a minimum number of trees and display all the splits whose support is above a predefined threshold. When the threshold is lower than.5, such a tree set has no consensus meaning.

To decide if a single consensus tree is acceptable of if several trees make a better representation, we introduce the *generalized score* criterion of an *X*-tree profile *π*:

### Definition 1

Let *P*_*π *_be a partition of *π* in *k* classes (*π*={*π*_1_,…,*π*_*k*_}), containing respectively {*p*_1_,…,*p*_*k*_}*X*-trees and let {*C*_1_,…,*C*_*k*_} be the majority consensus trees corresponding to these sub-profiles. The generalized score of *P*_*π*_, denoted $W^{k}(P_{\pi })$ (or $W^{k}$ when there is no ambiguity) is the sum over the classes, of the weight of the consensus tree multiplied by the number of trees in each class.

$$W^{k}(P_{\pi })=\underset{i=1,\ldots ,k}{\sum }p_{i}\times W_{\pi_{i}}(C_{i}).$$

This index can be viewed as a voting process ; the *p*_*i*_trees of *π*_*i*_ designate *C*_*i*_ as their representative tree ; its weight is equal to $W_{\pi_{i}}(C_{i})$. Each one of the *p*_*i*_ trees has this weight and the *π*_*i*_ class counts for $p_{i}\times W_{\pi_{i}}(C_{i})$. So, the generalized score is the sum of the class weights. It is a quadratic criterion, since majority edges count for $W_{\pi_{i}}(C_{i})$ and for *p*_*i*_.

 Counted so, the generalized score of the profile considered as homogeneous –that is with a single class–, is $W^{1}=m\times W_{\pi }(C_{1})$ which is a reference value. If a partition of the profile in *k*>1 classes which gives a greater generalized score ($W^{k}>W^{1}$) exists, then we conclude that the profile is not homogeneous and also that the consensus tree is not unique since there are *k* groups of genes with their own consensus, leading to a partition of the profile with a set of consensus trees of greater score.

When $W^{m}$ is the largest value, it means that each gene has its own evolutionary history and so, the generalized score corresponds to the atomic partition *P*_0_on *π*. This case is denoted as the *atomic* consensus. Each class (singleton) has its own *X*-tree as consensus and each edge receives a majority weight equal to 1. Each tree gets its number of internal edges as weight, which is bounded by *n*−3.

$$W^{m}=\overset{m}{\underset{i=1}{\sum }}n_{i}\leq m\times (n-3).$$

So, maximizing the generalized score over the set of all the partitions of *π* (denoted P(π)) one can decide if *π*admits a single, a multiple or an atomic consensus which is certainly justified if there is no majority edge.

### Example 2

**Figure 3:**
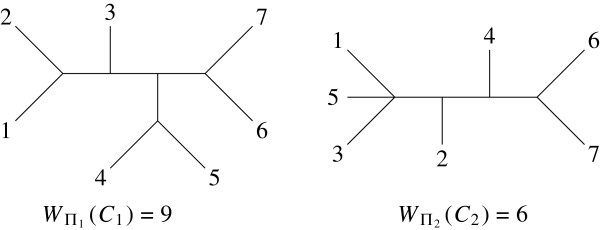
**Consensus trees *C***_**1 **_**and *C***_**2 **_**of classes *π***_**1 **_**and *π***_**2**_**.**

Consider profile *π*of Example 1, and the decomposition *π*_1_ = {*T*_1_, *T*_2_, *T*_5_} and *π*_2_ = {*T*_3_, *T*_4_}. *π*_1_ has one common bipartition (1,2,3 |4,5,6,7) and three majority ones, (1,2 |3,4,5,6,7), (1,2,3,4,5 |6,7) and (1,2,3,6,7 |4,5), which provide a consensus tree with weight 9. Trees in *π*_2_also share three majority (thus common) bipartitions, (1,3,5 |2,4,6,7), (1,2,3,5 |4,6,7) and (1,2,3,4,5 |6,7), making a consensus tree with weight 6. Both consensus trees are depicted in Figure 3.

So, the generalized score of profile *π*with its single consensus tree *C* is $W^{1}=5\times W_{\pi }(C)=5\times 7=35$. But the score resulting from decomposition *π*_1_|*π*_2_ is : $W^{2}=3\times W_{\pi_{1}}(C_{1})+2\times W_{\pi_{2}}(C_{2})=27+12=39$. It is also greater than the generalized score corresponding to the atomic partition of *π*, each tree *T*_*k*_ having a weight of 4, giving $W^{5}=5\times 4=20$.

### Proposition 1

Two *X*-trees admit a single consensus if and only if they have more than half their edges in common.

### Proof

Let *T*_1_ and *T*_2_ be two *X*-trees having *n*_1_ and *n*_2_ internal edges respectively. The generalized score of the two trees is $W^{2}=n_{1}+n_{2}$. If they share *k* edges, $W^{1}=2\times k$ and so $W^{1}>W^{2}$ if and only if $k>\frac{n_{1}+n_{2}}{2}$. □

One can formulate a similar result for *mX*-trees ; if there are *k* majority edges, $W^{1}\geq m\times k\times \lfloor \frac{m}{2}\rfloor$ and $W^{1}>W^{m}$ if and only if $k\times \lfloor \frac{m}{2}\rfloor >\frac{1}{m}\overset{m}{\underset{i=1}{\sum }}n_{i}$. One can conclude : for a profile of *m* resolved trees on *n* leaves, if $\lfloor \frac{m}{2}\rfloor >n-3$, one majority edge is sufficient to assert that $W^{1}>W^{m}$.

## Methods

Maximizing W over P(π) is not simple, since for each partition, the consensus tree of each class *π*_*i*_ or at least its weight, $W_{\pi_{i}}(C_{i})$ must be computed. As the number of classes is not fixed, we have developed two hierarchical algorithms to build a series of nested partitions with {*m*,(*m*−1),…,1}classes. For each one, its generalized score is computed and the partition within the hierarchy maximizing the score is retained. The atomic partition *P*_0_and the partition with a single class belonging to the series are competing. However, the best resulting score is not necessarily optimal over P(π).

### Average linkage hierarchical strategy

A classical approach in clustering consists in selecting a similarity function over the *X*-tree set and applying a hierarchical ascending method to build a series of nested partitions. We first recall two classical similarity measures between *X*-trees before using the average linkage algorithm (UPGMA) on one or the other similarity array. Other more sophisticated distances between trees [11] and other clustering algorithms, as in [9], can be used but the ones used here are fast enough to deal with hundreds of trees.

#### The *Robinson-Foulds (RF) similarity*

For any two trees *T*_*i*_ and *T*_*j*_ in *π*, each tree being considered as the set of its internal edges, the number of common edges is first computed. The *Robinson-Foulds* similarity, derived from their distance [12], is twice this number divided by the number of edges in both trees.

$$S(T_{i},T_{j})=\frac{2\times |\{a\in T_{i}\cap T_{j}\}|}{\left|T_{i}|+|T_{j}\right|}$$

#### The rate of common quartets (QS similarity)

The number of quartets in *X* having the same *topology* in two compared trees [13] are first counted. One similarity point will be assigned to quartet {*x*,*y*,*z*,*t*} if, in both trees, either at least one internal edge separates the same pairs (for instance {*x*,*y*} and {*z*,*t*}) or if they are both unresolved. Half a point is given when only one topology is resolved. If both are resolved and different, no similarity points are given.

##### Example 3

**Figure 4:**
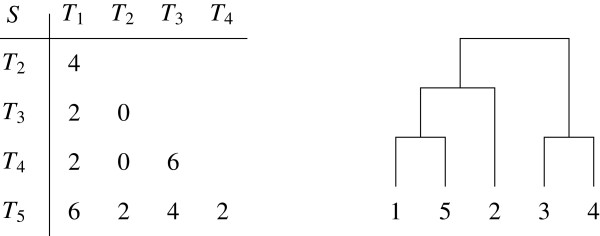
**The Robinson-Foulds similarity *S *without the common denominator (equal to 8) and its average linkage dendrogram.**

Coming back to the profile *π*in Example 1, the *Robinson-Foulds* similarity is given in the left hand table of Figure 4, ignoring the denominators equal to 8 since all the trees are resolved. One can start joining *T*_1_ and *T*_5_ or *T*_3_ and *T*_4_ since their similarity values are equal. In both cases, the same hierarchy (represented in the dendrogram on the right side of Figure 4) is obtained.

The nested partitions are (1|2|3|4|5), (1,5|2|3|4), (1,5|2|3,4), (1,2,5|3,4) and (1,2,3,4,5). The score values are $W^{5}=20$, $W^{4}=24$, $W^{3}=28$, $W^{2}=39$ et $W^{1}=35$, respectively. So, it is the partition in two sub-profiles, detailed in Example 2, which maximizes the generalized score giving two consensus trees for *π*.

### Merging the two classes maximizing the generalized score function

Given *k* classes making a running partition of the hierarchical process, to maximize the generalized score of a nested partition with *k*−1classes, one must join the two classes providing the best score value. Following this greedy principle, at each step, we evaluate the score value of all the fusions of any two classes and the two classes giving the maximum value are merged.

#### Example 4

Coming back to the profile *π*in Example 1, the score values of the successive tested partitions are given in the arrays of Table 2 corresponding to the successive steps. The left array is the initial table containing the values of partitions joining just two trees. It forms class {*T*_1_,*T*_5_} corresponding to consensus tree *T*_1,5_. The central array leads to merging classes *T*_2_ and {*T*_1_,*T*_5_}; Finally the last union merges *T*_3_ and *T*_4_, giving the same hierarchy as in Figure 4. At the first step, one could also make class {*T*_3_,*T*_4_} and the second step would be different since it leads to class {*T*_3_,*T*_4_,*T*_5_} giving another hierarchy and $W^{2}=35$ which is less than the previous choice.

**Table 2:** **The generalized score value of partitions of *π *merging two classes**

	***T***_**1**_	***T***_**2**_	***T***_**3**_	***T***_**4**_		***T***_**1,5**_	***T***_**2**_	***T***_**3**_		***T***_**1,2,5**_	***T***_**3**_
*T*_2_	20				*T*_2_	35			*T*_3_	28	
*T*_3_	16	12			*T*_3_	32	16		*T*_4_	28	39
*T*_4_	16	12	24		*T*_4_	26	16	28			
*T*_5_	24	16	20	13							

## Results and discussion

### On random trees

An heterogeneous profile *π*can generate very few majority edges, and consequently a consensus tree with a small weight when it is not reduced to a star tree. In this case, the generalized score of the atomic partition must be larger than the single consensus. We verified this, testing many sets of random *X*-trees ; they had no majority internal edge, and $W^{1}=0$. No proper class appeared and $W^{m}$ is always maximum.

In a more precise test, we first selected three rooted *X*-tree topologies with 16 leaves: the balanced binary tree (any subdivision is balanced and there are 8 cherries), the caterpillar tree (any subdivision is one taxon against the remaining ones, providing just one cherry) and a random topology obtained by random hierarchical subdivisions of the 16 leaves. For each topology we derived 30 trees, simulating DNA sequence evolution with only substitutions (3/4 transition, 1/4 transversion), avoiding the alignment process. For each tree a random root sequence is fixed and substitutions are randomly selected according to the random branch lengths for each tree. The sequences are 1000 nucleotides long and the substitution rate is.25, which means that, on average, 1/4 characters of the root sequence are changed in the terminal ones. The Kimura distance [14] between the 16 terminal sequences is computed and an *X*-tree is established using the NJ algorithm. So, for the three topologies, 30 trees are established.

We first verified that each consensus tree follows its initial topology and that the generalized score strongly supported a single consensus within the families. We then selected 10 trees from each topological set multiple times to make a profile clearly composed of three classes. The generalized score always recognized the three classes corresponding to the three topologies, and $W^{3}$ always gave the largest value.

### Homogeneous trees

We first tested trees computed by Brown et al. [15]. Their abstract states: *Here we use large combined alignments of 23 orthologous proteins conserved across 45 species from all domains, to construct highly robust universal trees. Although individual protein trees are variable in their support of domain integrity, trees based on combined protein data sets strongly support separate monophyletic domains. Within the Bacteria, we placed spirochaetes as the earliest derived bacterial group. However, elimination from the combined protein alignment of nine protein data sets, which were likely candidates for horizontal gene transfer, resulted in trees showing thermophiles as the earliest evolved bacterial lineage.*

Since possibly divergent proteins have been eliminated, the single consensus must be strong and give the largest score. In fact, there are 22 majority internal edges over the 333 present in the 23 trees (with 45 species there are at most 989 bipartitions). The consensus tree has a weight of 430, revealing that each edge is supported by nearly 20 trees, so they are strongly majority, and the generalized score is $W^{1}=9890$. The atomic consensus gives $W^{23}=964$. Decreasing the number of classes increases the scores, but they never reach the single consensus tree value. The best secondary value is obtained for 2 classes, isolating one singleton and giving $W^{2}=8673$. It is, therefore, confirmed : there is a single consensus tree for this homogeneous tree set.

When the profile *π*is homogeneous, the single consensus must give the largest score. It is what is expected from bootstrap trees corresponding to a single gene ; we first verified this on trees corresponding to *Escherichia Coli* strains.

### 9 genes on 30 *E. coli* strains

In a previous work with P. Darlu, we asked the same question of how to recognize divergent genes sequenced on the same *X* taxa set. We proposed a new method, *TreeOfTrees*[16], establishing a tree of which each leaf corresponds to a single gene (in fact bootstapped trees of this gene) and each internal edge receives a robustness coefficient allowing the separation of subsets of trees that are topologically different. It was the first attempt to statistically evaluate whether two trees are significantly closer to each other than to a third one. This method has been proved efficient on both simulated and real data.

The application was done on 9 genes (DNA sequences) in 30 strains of *Escherichia coli*[17]. Let *X* be the strain set and *G* the set of genes corresponding to 6 housekeeping genes (icd, pabB, polB, putP, trpA, trpB), plus 3 others, HPI, DR and UR (High Pathogenicity Island and its Downstream and Upstream regions), which are known to have been transferred. The corresponding sequences were first aligned and 500 bootstrap trees were obtained with PHYML [18].

**Figure 5:**
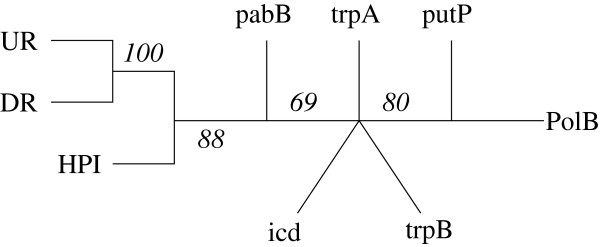
**The final tree on *E. coli *genes given by the *TreeOfTrees *method.**

The *TreeOfTrees* method is based on the comparison of these bootstrap trees. At each iteration, corresponding to one bootstrap step, the algorithm compares |*G*|*X*-trees, computing a distance between them and using a distance method (NJ) to define a *G*-tree, i.e. a tree whose leaves are the genes. At the end of the 500 iterations, 500 *G*-trees make a profile and a consensus tree is established indicating a bootstrap value for internal edges, as usual. When this value is high, one can deduce that the genes on both sides of this edge correspond to different gene tree sets, revealing different topologies. With these 9 genes, we have obtained the *G*-tree depicted in Figure 5 with bootstrap values displayed on the edges.

Based on these values in the consensus *G*-tree, we conclude that the *X*-trees (on the *E. coli* strains) built from the HPI, UR and DR sequences are significantly different from the others. The biological interpretation is discussed in Schubert et al. [17]. Before continuing with this data set, we would like to underline that the *TreeOfTrees* method does not make it possible to separate a single gene since the robustness coefficients are only defined for internal edges.

First, we have computed a consensus *X*-tree for each gene. The first thing to verify is that any 500 bootstrap tree set constitutes a homogeneous profile admitting a single consensus tree. This can be observed in Table 3 indicating, for each gene, the number of total bipartitions, the number of majority bipartitions, the weight of the consensus tree, the corresponding generalized score $W^{1}$, the number of classes of the best partitioning of the profile in more than one class, (with the number of elements of the other classes) and its generalized score.

**Table 3:** Results on bootstrap trees

	***BiP***	***Maj***	***W***	$W^{1}$	***NbClas***	$W^{m}$
UR	8	7	2623	1311500	2	1304768
trpB	28	15	6248	3124000	2	3114271
trpA	45	9	3824	1912000	3	1900390
putP	57	17	6608	3304000	2	2508400
polB	119	14	5331	2665500	2	2639187
icd	69	15	5681	2840500	2	2929008
HPI	76	13	4971	2485500	2	2467626
pabB	57	8	3667	1833500	2	1827846
DR	12	8	2685	1342500	2	1335146

For each set of bootstrap gene trees, the number of bipartitions (*BiP*), the number of majority bipartitions (*Maj*), the weight of the consensus tree (*W*), its generalized score ($W^{1}$), the number of classes of the best multiple consensus (*NbClas*) and the generalized score of this partition ($W^{m}$) are indicated.

For all the genes, except icd, $W^{1}$ is maximum. The competing partition has 1 or 2 extra classes which are very small ; gene putP is an exception since the second class has 80 elements, but $W^{2}\ll W^{1}$. For icd, $W^{2}>W^{1}$ but the two values are very close and the optimal partition has only one other class with 4 elements sharing 8 common bipartitions. So, the profiles generated by bootstrapping are homogeneous and therefore recognized by the generalized score function.

These 9 consensus trees make our last profile. It generates 99 bipartitions, 3 of them being majority. The best generalized score obtained by the average linkage algorithm applied to the Robinson-Foulds similarity is shown on the first row of Table 4, and the quartet similarity on the second. The third row contains the generalized scores given by the second algorithm.

**Table 4:** **Generalized scores of the *E. coli *genes for all possible numbers of classes**

**NbClas**	**1**	**2**	**3**	**4**	**5**	**6**	**7**	**8**	**9**
*AL*(*RF*)	144	150	174	147	154	139	120	130	140
*AL*(*QS*)	144	150	135	159	169	136	146	129	140
*GA*	144	168	**182**	147	160	145	155	130	140

The two first rows correspond to the average linkage (*AL*) algorithm on both similarity indices and the third one corresponds to greedy algorithm (*GA*) merging the two classes maximizing the score function.

As can be seen, $W^{1}$ is larger than $W^{9}$, and the single consensus is better than the atomic one. But the single consensus tree score is greatly improved by the partition in 3 classes composed of {HPI, UR, DR}, {pabB, trpA, trpB, icd, PolB} and {putP}. It is compatible with the *G*-tree of Figure 5 in which {putP} cannot be separated. The best partition in 2 classes places {HPI, UR, DR, putP} apart from all the others ; its generalized score value (168) is greater than $W^{1}$ but the optimal partition does not recognize class {UR, DR}. This closeness in Figure 5 may be due to the fact that UR and DR *X*-trees have a very low resolution, as do the whole set of bootstrap trees, since over 30 taxa, only 8 to 119 bipartitions can be observed.

## Conclusion

We have described a simple and efficient method to decide if there is a single consensus between trees or not, and to establish a partitioning method that detects divergent genes. Applying a clustering hierarchical algorithm, the optimal partition is not certified. But it is sufficient to find a partition with a generalized score greater than $W^{1}$ to assess the divergence of the profile and to search a decomposition in disjoint classes.

What remains, therefore, is to compare the consensus trees of classes in order to explain the divergence, suspected paralogy or possible transfers. More generally, the few, if any, discordant trees, can be removed to keep only genes that share the same evolutionary history and reflect the real tree of species. This method should also be extended to profiles made of trees connecting different taxa sets. The consensus tree notion must first be enlarged before combining trees connecting different subsets of *X*.

## Endnote

^a^For instance, an *X*-tree computed from the sum of the unitary tree distances [19] which can be denoted as the average tree; the NJ-tree of this distance between trees of Figure 1 is identical to *C*_*E*_.

## Competing interests

I am an employee of the french “CNRS” providing the article-processing charge and no other organization. There is no other financial or non-financial competing interests in relation to this manuscript.
